# Listening to Voices in Appalachia Gathering Wisdom from the Field about Substance Abuse Recovery Ecosystems

**DOI:** 10.13023/jah.0203.11

**Published:** 2020-07-19

**Authors:** Bruce Behringer

**Affiliations:** Department of Family Medicine, East Tennessee State University

**Keywords:** Appalachia, drug abuse, opioids, recovery, rural communities, mortality rate, overdose

## Abstract

New qualitative data collected through six regional community-based listening sessions and community meetings are presented that describe elements of the Appalachian Regional Commission’s Recovery Ecosystem Model. These data informed the Model, which was used in formulating the new ARC Recovery-to-Work initiative. Input was intentionally solicited from multiple sectors, including persons recovering from substance abuse disorder, treatment and recovery service providers, workforce development agencies, employers, and community advocacy groups.

## BACKGROUND

Numerous studies have identified higher opioid overdose mortality rates in the Appalachian region than in the non-Appalachian parts of the country.^1^–^3^ Multiple parts of the region lay claim to being “ground zero” for this current iteration of the nation’s substance abuse crisis.^4^–^6^ Beyond high mortality rates lie an estimated but unmeasured population of addicted individuals whose participation in the workforce is lost.^7^ The age cohort most affected is younger and includes those in their prime working years. This lost workforce creates a significant challenge to economic development in Appalachia’s rural communities.

Feedback from communities and numerous ARC studies indicates the importance of employment in an individual’s recovery process from substance abuse. However, a review of literature and public reports has found little about the interrelated issues of substance abuse disorders, recovery, and workforce reentry. To address this gap, the Appalachian Regional Commission (ARC) organized community meetings in six states to identify elements of effective recovery ecosystems that would help individuals maintain recovery while taking steps to achieve employment. The meetings provided valuable input to guide development of future ARC programs. A full description of the process of organization and results of these meetings has been reported.^8^ This manuscript is intended to describe new primary source data collected through the aforementioned community-based meetings about the subject.

The meetings were a proactive means of learning from the wisdom of the field. Input was solicited from individual and community voices. These included stories from persons in recovery relating their experience about maintaining recovery while seeking training and employment, insights from state agencies and their local offices about existing policies and initiatives, hiring difficulties faced by employers, and lessons learned from recovery services personnel. This mix of voices added an understanding of many complex personal and service system challenges that ARC would be required to consider in promoting a healthier workforce to spur regional economic improvement.

Input from the meetings was part of a four-step yearlong process to develop ARC’s response to this issue, the Recovery to Work Initiative. Key elements of an ideal recovery ecosystem designed to assist individuals with substance abuse disorder (SUD) back to employment were identified and a working model was created. The regional meetings identified issues and validated individual and system challenges. A new Substance Abuse Advisory Council reflected on this input, considered current practices and policies, and generated recommendations for action. ARC then turned these recommendations into funding programs to support recovery ecosystems. A full description of this four-step process to create the ARC Recovery to Work initiative is published concurrently in the *Journal of Appalachian Health*.^9^ This article describes the second of four steps: regional listening sessions and public meetings.

## METHODS

**Figure 1:**
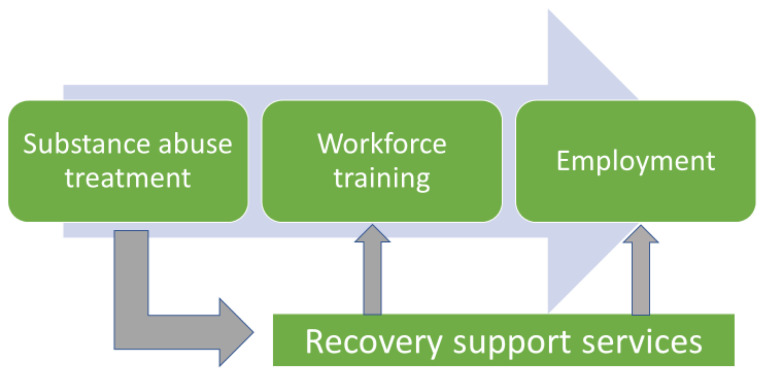
The ARC Recovery Ecosystem Model

Appalachian Regional Commission staff developed the Recovery Ecosystem Model (the Model) prior to organizing regional meetings. The Model (Figure 1) displays an idealized flow of persons following substance abuse treatment. Three key post-treatment elements are defined: workforce training, employment re-entry, and continuous recovery support services. Input was collected through the meetings to describe characteristics and requirements for each Model element. Meeting input was also used to identify challenges faced by communities in operationalizing a successful local recovery ecosystem. ARC contracted with a single moderator to facilitate all meetings. The moderator used the Model to assure continuity across all six sessions.

### The Listening Sessions and Public Meetings Process

Six meetings were organized between December 2018 and April 2019. Assistance was provided to ARC by ARC state alternates, local development districts (LDD), congressional offices, and other partners. Meetings were located in the Appalachian regions of six states: Virginia (Big Stone Gap), North Carolina (Wilkesboro), Alabama (Muscle Shoals), Kentucky (Pineville), Ohio (Portsmouth) and West Virginia (Beckley). State partners and LDDs selected public locations with easy access and parking at five community colleges and one state park. The partners assumed responsibility for local logistics, assisted in speaker and participant recruitment, and distributed ARC public press notices to regional media and organizations.

The Appalachian Regional Commission actively integrated input from three important groups into meeting planning to guide formulation of Model. First, persons affected by substance abuse spoke at each listening session, sharing personal stories of their post-treatment journeys to recovery, job training, and employment. Second, state government offices charged with aspects of substance abuse and economic development were engaged. Participants included representatives state departments of community development, commerce, workforce, public health, and drug control. Third, representatives from local and regional service organizations and community leaders were recruited to participate, including community colleges, rural primary care centers, foundations, employers, addiction recovery organizations, regional employment training programs, law enforcement, criminal justice, and local government.

Each meeting day followed a common agenda. The morning was called a listening session. A public meeting was conducted in the afternoon in the same location. Five of six states followed this approach. Alabama conducted a multi-sector roundtable sponsored by the region’s member of Congress; this event combined the listening session and community meeting elements.

Participation in the listening session was by invitation. Stakeholders were identified in the community to ensure that diverse sectors were represented. ARC Federal Co-Chair Tim Thomas introduced the listening sessions and provided background. Additional welcome and overview comments were provided by a state official. A local individual in recovery then shared a personal story of the journey following treatment to recovery and the challenges encountered in securing training and meaningful employment. Following the presentations, participants rotated among three stations in small groups, recording insight and ideas for each of the three post-treatment Model elements on flip charts: recovery services, workforce training, and employment. ARC staff acted as flip chart recorders and subsequently reported findings to the full audience for discussion. The Federal Co-Chair and state official provided reactions and summaries. Average listening session attendance was 25 people.

The afternoon public meeting opened with introductions and comments from the ARC Federal Co-Chair and a state official. A panel of area speakers presented insights about local, regional, and state issues for each of the three Model elements. The audience contributed additional ideas during a facilitated public discussion. Average public meeting attendance was 75 people. Several members of Congress, and multiple state and local elected officials also attended the public meetings.

### Data Collection

Three methods were employed to gather ideas from participants. First, participant ideas from the morning listening session were recorded on flip chart pages. Prepared questions guided discussion at each Recovery Ecosystem Model flip chart group. Second, ARC staff and the facilitator recorded field notes during the discussion periods of both morning and afternoon sessions. Third, all participants attending the afternoon public meetings were invited to record ideas on index cards by responding to the statement, “The most important recommendation I would make to ARC about designing and planning initiatives to help adults with substance abuse disorder secure meaningful employment following treatment is….”

All flip chart contents, field notes, and index card ideas were transcribed. Ideas generated from the three sources were assigned to one of the three Recovery Ecosystem Model elements. Similar ideas were combined, resulting in organically formed broad themes and specific sub-themes. For example, one workforce training theme was the content of training and job placement services, and policies that allow medication assisted therapy services to be offered during training hours was a sub-theme. Upon completion of all six meetings, all states’ themes, sub-themes, and idea counts were combined and reanalyzed to ensure consistency of language across the states’ reports.

## RESULTS

### The Voices of People In Recovery

Individuals in recovery provided personal accounts of the post-treatment situations summarized in the Recovery Ecosystem Model. Each speaker acknowledged multiple personal challenges following treatment, including finding local recovery services. Speakers reiterated that recovery is frequently not a linear process. Relapse to substance abuse and restarting the recovery process is frequent. One speaker noted:

I would like to say something. I’m hearing something from certain people who work with people with substance abuse say that relapse is part of recovery. Relapse is not part of recovery. Like I say, a lot of people relapse. But relapse is a part of dying.

Sustaining recovery begins with a realistic assessment of each individual’s situation and identifying the combination of supports needed. A speaker at another state meeting reported:

Probably the hardest thing about this disease is figuring out each person’s fear and anxiety after stepping out of treatment and trying to figure out what am I supposed to do next.

Individuals in recovery recounted similar challenges led by lack of transportation, finding housing, continuing legal difficulties, childcare needs, and lack of cash. However, there was another broad theme, that of recovering a personal sense of purpose, and a desire to escape the substance abuse lifestyle. Separate speakers confirmed this intention:

I’m not only recovering from substance abuse; I’m recovering from the lifestyle I became comfortable with while I was in the grips of that substance use. I didn’t feel a part of society. And the main reason I probably didn’t feel a part of it was because I was damaged. I didn’t know how to be a productive person.

A man or a woman without a purpose is lost. [Recovery services] try to keep them going forward, which gives them goals, motivation, and a sense of self-accomplishment—that’s all I ever wanted my whole life was to have a purpose.

All speakers spoke consistently about the importance of work and the fact that a job provides a sense of purpose, which is critical to their recovery:

Recovery and work provided that sense of accountability that I had not had for some time.

Employment was such a key piece for me. It built my self-esteem and it gave me confidence that [someone] believed in and invested in me. A job was the avenue for me to be able to network and build relationships. Employment helped to show what sobriety looks like and learn to love myself.

Without employment, we will fall back into the same thing that once killed us. We suffer from a disease which is truly fatal, but 100 percent treatable.

Finding a job, however ready a person in recovery may be, is not certain. Each speaker noted stigma against substance users, each in a different way:

You’re recovering from a hopeless state of mind and body and you go out there and you beat the bushes for a job and you’re trying to get your feet back on the ground. These employers need some incentive to hire somebody that has a substance abuse problem. It’s just so frustrating to people when they’re trying to get their lives straightened out, provide for their families, and they get turned down, and turned down, and turned down. So one reason that some of the folks may not relapse is they can find employment of some kind. Gives them hope.

Each speaker talked about finding the one person who was supportive and provided guidance. For one, it was the very judge whose sentence led to an out-of-town treatment referral. For another, it was a local primary care physician who wrote a letter to prospective employers to vouch for persistence in recovery. For still another, it became fellow members of the local recovery community who helped the individual though the steps to get services and make better decisions, leading the individual to becoming a productive community member.

### Themes Based on the ARC Recovery Ecosystem Model

Table 1 summarizes the 965 ideas generated through the six listening sessions organized into themes and sub-themes for the Recovery Ecosystem Model elements. More ideas were gathered about recovery services (58%) than for employment (25%) and workforce training (17%). A full compilation of ideas about each element is summarized in a comprehensive list.^10^

By far, the largest number of ideas across all state meetings focused on needed support services. This directly addressed the Federal Co-Chair’s request to identify services required to develop effective ecosystems. Table 1 includes themes that affirmed the importance of continuous recovery services throughout the process displayed in the Model. Important sub-themes emerged: affordable, sober, and safe transitional housing, often with in-house treatment services; services for family needs like child and elder care; financial literacy and planning; and help with credit score recovery, domestic violence recovery, legal aid, and access to health care. Transportation issues were identified as the top barrier in pursuing and maintaining recovery, training, and work. Many rural areas lack public transportation. Loss of a driver’s license due to court action or inability to pay fines prevents individuals in recovery from accessing key services. The lack of a locally organized infrastructure of services to support those in recovery was another consistent sub-theme. Other suggestions for supporting individuals in recovery included peer-to-peer support between people in recovery to identify and access services; recovery mentors in the workplace; success coaches; and case managers.

Beyond the personal needs of individuals in recovery, the meetings identified important system issues. To promote success, participants agreed that a recovery ecosystem needs a community-involved design process built on a standard continuum of care. Services would be organized as an ecosystem with established interorganizational linkages and service handoffs. Community members involved in an ecosystem development process would review local problems and develop plans that integrate the combined perspectives of law enforcement, education, health, treatment and recovery services, training programs, and employers. Multi-sector input would promote communication, formalize structures, and develop protocols. Several existing ecosystem organizing strategies were identified, including one-stop multiservice locations operating with a “no wrong door” philosophy. Examples of services integration included colocation, shared personnel, and interactive service information systems. Representatives from several locales cited practices that effectively integrate housing with wrap-around social services and job training in single locations.

That fewer ideas were generated about workforce training did not reflect disinterest. Participants instead clarified that while job training is an important step within the recovery ecosystem, those in recovery must first be ready. A consensus concern was the general absence or lack of confidence in life skills, including soft skills, among those in recovery, and the impact this deficit has in enrollment and retention in training programs. While vocational training and technical skills are important to enter the workforce, basic job reentry skills are also needed, such as how to complete an application, interview training, and reorganizing one’s life to meet work, family, transportation, and financial management necessities. Because many persons in recovery are without high school diplomas, training should begin with adult education and GED access. Participants suggested integrated work experience, on-the-job training, and job coaching with mentoring to reinforce the sense of purpose for maintaining recovery. Success was greatest when training and recovery services were linked through colocation, using agreements with transitional housing organizations and medication-assisted therapy service providers.

Participants recognized that programs to help those in recovery transition back into the workforce require more individualized attention, a broader array of wrap around services, and longer timelines to accomplish objectives. Participants cited the following as the keys to personal success: the development of individualized plans, maintaining continuous contact through counselling, formal case management, and informal peer support.

A focus on employment was seen as an important ecosystem outcome. Success was seen as contingent on improving connections between training and employers. Many ideas were proposed to address employers’ fears of the social costs of public perception and community standing if it becomes known that they hire those in recovery. Employers often have concerns about the recovering individual’s mental health, honesty, dependability, and potential for turnover, even for potential candidates who have required skills. Stigma is often encountered by employees, especially those with criminal records. Human resources policies and lack of personal support from existing workers contribute to employers’ hesitancy to hire those in recovery. A general lack of understanding of addiction and recovery feeds beliefs that form barriers to job placement. Real success stories are needed. So, too, are compilations of best practices, including sample human resources policies and work practices designed to retain those in recovery.

Participants offered multiple similar examples of financial incentives to employers to support hiring people in recovery. Innovative state strategies included work opportunity tax credits, subsidies tied to hiring, paid internships with further job training opportunities, programs to protect employers against financial risk by addressing liability concerns, and fidelity bonds for employers.

Several ideas promoted cooperative regional planning approaches to engage community leaders, workforce agencies, educators (including community colleges), and employers. Support was proposed for personnel who build bridges between employers, workforce development agencies, and workers already in recovery (such as peer counselors). Overall, recognition of issues relating to substance abuse, recovery, and workforce re-entry was seen to be pivotal in changing prevalent community-wide attitudes and barriers. Participants discussed examples of ways to introduce those in recovery to employers. Local plans should promote networking, steps to match job candidates with the right opportunities, and ways to ensure pathways for local, meaningful employment that pays a livable wage and offers adequate hours. This type of regional planning could access and coordinate available state and federal funding streams.

### Commonalities and Differences of Themes Across States

The use of active facilitation during listening sessions enabled participants to identify and elaborate themes unique to each state. Virginia sessions emphasized detailed elements of a recovery ecosystem and need for regional communication and cooperation. North Carolina focused on the value of volunteers, employer needs, and multi-community regional efforts. Kentucky emphasized public certifications for services, formal interorganizational linkages, and the lack of a central source for best practices. Ohio participants stressed a systems approach to coordination, employer engagement, and acute housing and transportation needs. West Virginia highlighted the need for a local focus, community service options, and engaging people in recovery as assets. Alabama primary themes included care coordination, adoption of a continuum of care approach, and design of targeted marketing campaigns. The minor agenda modification in Alabama did not result in significant differences in input compared to other states. When differences in emphasis across states were pointed out, attendees traced differences to variations in the intensity of local problems, immediacy and visibility of the substance abuse issues in the community and media, and length of history of community attempts to address the issue.

Several common themes were discovered across states that might act as guideposts for future action:

- The success of organized recovery ecosystems seems contingent on visible community desire and commitment to overcome stigma and engage residents in recovery in the workforce.
- Cross-sector communication is important to identify, interpret, and discuss solutions for the complex issues faced by those in recovery seeking workforce re-entry.
- Coordination is required at the local level between multiple programs to encourage successful partnerships. Local coordination is pivotal for effective use of federal and stated resources.
- The ARC Recovery Ecosystem Model defines employment as a system outcome and helps focus individual goals across multiple programs and agencies.
- Persons in recovery represent a potential untapped resource in the region’s workforce.
- Peer counselors who are in recovery have become assets to others through being employed by recovery service organizations, training programs, and employers.
- ARC’s attention to defining elements and flow of the Recovery Ecosystem Model is an important validation of the need for a comprehensive community-oriented approach.

## CONCLUSIONS

To our knowledge, no other federal agency has focused on substance abuse recovery ecosystems that emphasize workforce reentry. While Appalachian needs may not be unique, the listening sessions were very helpful in verifying acute regional concerns. The meetings identified the need for broad-based, multisector recovery ecosystems organized by communities. A successful recovery ecosystem was seen to have many positive and measurable personal, business, and community impacts.

Participants confirmed that linking the three key recovery ecosystem elements within a single model with workforce reentry as a stated goal was an important step forward. The Model places all key stakeholders on a single page—persons in recovery, substance abuse treatment providers and community recovery programs, workforce development agencies, and employers. The listening sessions identified multiple best-practice services but not a fully developed recovery ecosystem. Examples of cooperation were found between community organizations and service providers to creatively form networks to build local and regional organizational commitments. Some of these efforts have been further profiled for sharing with other communities.^11^ Samples of assessments, policies, convening approaches, formal linkage agreements, evaluation measures, and insightful community stories need to be shared to successfully begin developing recovery ecosystems.

The Appalachian Regional Commission’s mission, structure, and history facilitated this comprehensive approach. State agencies affiliated with ARC and LDDs helped to accomplish the efficiently organized set of local meetings. ARC and state agencies built on contacts garnered over decades to effectively convene local meetings and gather input about the Recovery Ecosystem Model.

Data analysis uncovered suggestions that attention is required in attending to the unrecognized steps between the Model elements. This included concern about steps to prevent those in recovery from “falling between the cracks.” Five additional sub-steps could be added to benefit future versions of the Model: (1) treatment organizations’ handoffs to recovery services; (2) treatment and recovery services coordination with workforce training programs; (3) linkages between workforce training programs with selected employers especially designed for employment for those in recovery; (4) integration of recovery services by employers for employees in recovery; and (5) broadly defined collaborative infrastructure interventions that organize and manage ecosystem operations.

By proactively seeking wisdom from the field, ARC has advanced an understanding of a regional issue that combines the interests of the health and economic sectors, which have not typically worked together on this issue at the community level. The ideas generated in six state meetings reinforce the need for multisector interventions rather than a series of categorically-defined programs. It is acknowledged that meetings were conducted in only six of thirteen states that make up Appalachia. ARC conducted a subsequent review of findings by an advisory council with representatives from all states to confirm generalizability of the results. The complex problems explored and the wealth of ideas gathered underscore the importance of the role of local partners. Recovery ecosystems will require a combination of resources from regional, state, and federal sources to effectively help individuals to move form recovery to work.

## Figures and Tables

**Table 1:** Ideas Organized into Themes Using Elements in the ARC Recovery Ecosystem Model

	Percent of all ideas	Ideas from six state meetings
**Total ideas**		**965**
**Workforce training**	**17**	
Content of job training and placement services		59
Training and placement plans for individuals		32
Job training resources		20
Factors influencing links between training and placement with employers		54
**Employment**	**26**	
Regional approach to organizing a market for job opportunities		57
Address employer needs		136
Factors to be addressed in fitting candidates in recovery with available jobs		53
**Recovery support services**	**57**	
Services needed to support recovery ecosystem		180
Factors moving into recovery		96
Program characteristics to promote success		80
Linkages and handoffs		68
Immediate post-treatment recovery service needs		67
Actions to promote linkages and handoffs		63
